# Characterization of four novel bacteriophages targeting multi-drug resistant *Klebsiella pneumoniae* strains of sequence type 147 and 307

**DOI:** 10.3389/fcimb.2024.1473668

**Published:** 2024-10-04

**Authors:** Greta Ponsecchi, Tommaso Olimpieri, Noemi Poerio, Alberto Antonelli, Marco Coppi, Gustavo Di Lallo, Mariangela Gentile, Eugenio Paccagnini, Pietro Lupetti, Claudio Lubello, Gian Maria Rossolini, Maurizio Fraziano, Marco Maria D’Andrea

**Affiliations:** ^1^ Department of Biology, University of Rome “Tor Vergata”, Rome, Italy; ^2^ PhD Program in Evolutionary Biology and Ecology, Department of Biology, University of Rome “Tor Vergata”, Rome, Italy; ^3^ Department of Experimental and Clinical Medicine, University of Florence, Florence, Italy; ^4^ Microbiology and Virology Unit, Florence Careggi University Hospital, Florence, Italy; ^5^ Nutrition, combating infection and Antibiotic Resistance in Rehabilitation (NARR) Joint Laboratory for Antimicrobial Resistance Research and Control, University of Florence-Istituto di Ricovero e Cura a Carattere Scientifico (IRCCS) Don Gnocchi Foundation, Florence, Italy; ^6^ Department of Life Sciences, University of Siena, Siena, Italy; ^7^ Department of Civil and Environmental Engineering (DICEA), University of Florence, Florence, Italy

**Keywords:** phage, *Klebsiella pneumoniae*, multi-drug resistance, phage-therapy, *Klebsiella pneumoniae* ST147, *Klebsiella pneumoniae* ST307, carbapenem-resistance

## Abstract

The global dissemination of multi-drug resistant (MDR) pathogenic bacteria requires the rapid research and development of alternative therapies that can support or replace conventional antibiotics. Among MDR pathogens, carbapenem-resistant *Klebsiella pneumoniae* (CR-Kp) are of particular concern due to their extensive resistance profiles, global dissemination in hospital environments, and their major role in some life-threatening infections. Phages, or some of their components, are recognized as one of the potential alternatives that might be helpful to treat bacterial infections. In this study, we have isolated and characterized four lytic bacteriophages targeting *K. pneumoniae* strains of Sequence Type (ST) 307 or ST147, two predominant high-risk clones of CR-Kp. Phages, designated vB_KpS_GP-1, vB_KpP_GP-2, vB_KpP_GP-4, and vB_KpP_GP-5, were isolated from sewage wastewater samples. The vB_KpS_GP-1 phage was a siphovirus unable to establish lysogeny with its host, while the other three were podoviruses. While 85.7% of *K. pneumoniae* strains of ST307 were selectively lysed by the phages vB_KpS_GP-1 or vB_KpP_GP-5, the other two phages were able to lyse all the tested strains of ST147 (*n* = 12). Phages were stable over a broad pH and temperature range and were characterized by burst sizes of 10–100 plaque forming units and latency periods of 10–50 minutes. Genome sequencing confirmed the absence of antibiotic resistance genes, virulence factors or toxins and revealed that two phages were likely members of new genera. Given their strictly lytic nature and high selectivity towards two of the major high-risk clones of *K. pneumoniae*, cocktails of these phages could represent promising candidates for further evaluation in *in vivo* experimental models of *K. pneumoniae* infection.

## 1 Introduction

The spread of multi-drug resistant (MDR) bacterial pathogens in hospital environments represents one of the leading contemporary public health threats that requires global and coordinated actions (Centers for Disease Control and Prevention, 2019; World Health Organization, 2021). Indeed, infections caused by MDR bacteria are frequently associated to high mortality rates, prolonged hospitalizations, and increased healthcare-associated costs, factors that translate, for example, to estimates of a global burden of 1.27 million deaths in 2019 alone (Giske et al., 2007; Murray et al., 2022).

Among MDR Gram negative bacteria, carbapenem-resistant *Klebsiella pneumoniae* (CR-Kp) are one of the major clinically relevant pathogens (Navon-Venezia et al., 2017; Karampatakis et al., 2023), reaching rates higher than 20% in several countries (Di Pilato et al., 2024). For these reasons, CR-Kp are considered as a critical priority antibiotic-resistant pathogens for which new antibiotics are urgently needed (Tacconelli et al., 2018; World Health Organization, 2024).

The rapid spread of CR-Kp in clinical settings is largely attributed to the global dissemination of a restricted number of successful clones, which have been the cause of several outbreaks at global level (Navon-Venezia et al., 2017; Dong et al., 2022; Karampatakis et al., 2023). These clones, part of the so-called “high-risk clones” (HiRiCs), belong to few different Clonal Groups (CG), including CG11/CG258, CG15, CG37, CG101 and, more recently, ST395, CG307 and CG147 (David et al., 2019; Wyres et al., 2019; Peirano et al., 2020; Shaidullina et al., 2023). HiRiCs pose a significant threat to human health, considering their large dissemination and the extended antibiotic resistance levels, which can also include drugs recently approved for clinical uses. Due to the limited availability of effective antimicrobial compounds and the slow progress in the development of new antibiotics, novel therapeutic strategies to address this growing crisis are therefore urgently required.

Bacteriophages have emerged as a promising approach to tackle antibiotic resistant bacteria. Phage therapy is an old antimicrobial strategy proposed more than a century ago that currently aims at using lytic bacteriophages for the treatment of bacterial infections (Gordillo Altamirano and Barr, 2019; Kortright et al., 2019; Strathdee et al., 2023). In the context of the global dissemination of a restricted number of MDR clones, as it has been observed with CR-Kp, bacteriophages appear of particular interest as a strategy to selectively target and kill bacterial pathogens.

The current study reports on the isolation and characterization of four newly discovered lytic bacteriophages active against *K. pneumoniae* isolates of Sequence Type (ST) 147 and ST307.

## 2 Materials and methods

### 2.1 Bacterial strains

Four *K. pneumoniae* clinical strains representative of two major HiRiCs, namely ST147 and ST307, were used as indicators for the isolation of lytic bacteriophages. Three out of four isolates were carbapenem resistant due to the production of different carbapenemases, while the remainder was a member of ST307 negative for such enzymes (**Table 1**). The ability of the isolated phages to target different clones of *K. pneumoniae* was assessed by using a collection of CR-Kp clinical strains previously characterized in Italian and European surveillance surveys on the spread of carbapenem-resistant *K. pneumoniae* (Cannatelli et al., 2013; Giani et al., 2013; D’Andrea et al., 2014; David et al., 2019; Di Pilato et al., 2021; Henrici De Angelis et al., 2021; Coppi et al., 2022; Di Pilato et al., 2022). Bacteria in this collection have been selected with the aim to choose clinical isolates belonging to high-prevalent STs, producing epidemiologically relevant carbapenemases and characterized by the maximum genetic distance as resulting by whole-genome comparisons.

**Table 1:** Characteristics of the clinical strains used for the isolation of the four bacteriophages.

Strain ID	Isolated phage	Carbapenemase	Collection date	ST	Reference
EuSCAPE_IT395	vB_KpS_GP-1	–	04/01/2014	307	David et al., 2019
KP411	vB_KpP_GP-5	KPC-2	29/06/2016	307	Di Pilato et al., 2021
KP263	vB_KpP_GP-2	KPC-3	13/05/2016	147	Di Pilato et al., 2021
KP-20LU	vB_KpP_GP-4	NDM-1	04/01/2019	147	Di Pilato et al., 2022

### 2.2 Phages isolation

Bacteriophages were isolated from samples of untreated wastewater collected near three hospitals located in central Italy using standard enrichment followed by double-layer agar overlay methods. Briefly, an aliquot of 4.8 ml of each sample was mixed with 5 ml of double-concentrated Lysogeny Broth medium (LB; tryptone 10 g/L, NaCl 10 g/L, yeast extract 5 g/L, pH=7.0) and with 200 µl of a bacterial inoculum of the indicator strain grown overnight (O/N) in LB. The suspension was subsequently incubated O/N at 37°C under shaking and centrifuged at 1,500 x *g* for 20 min. The obtained supernatant was then filtered through 0.45 µm and 0.22 µm Minisart^®^ filters (Sartorius Minisart, Sarstedt, Italy) to remove bacterial cells and sample debris and used for screening the presence of bacteriophage by spot-test, as previously described (Kutter, 2009). Briefly, 200 μl of a O/N culture of each indicator strain was mixed with 5 ml of soft agar (LB + 0.5% w/v agar), then poured onto LBA plates (LB + 1.5% w/v agar) to form a bacterial lawn. Five microliters of tenfold serial dilutions of each enriched suspension were spotted on the lawn and plates were incubated at 37°C O/N. After incubation, spots having plaques with clear lysis zones were considered positive for the presence of bacteriophages. Well-isolated plaques were then picked and suspended in SM buffer (100 mM NaCl, 8 mM MgSO4•7H2O, 50 mM Tris HCl pH = 7.5). For each phage, three rounds of infection followed by single-plaque isolation have been performed to obtain pure bacteriophage suspensions.

### 2.3 Large-scale production of bacteriophage suspensions and purification

Propagation of isolated phages obtained by using the EuSCAPE_IT395, KP411, and KP-20LU strains was performed using double-layer agar overlay as reported previously (D’Andrea et al., 2017), while phage selected on the KP263 strain was obtained in liquid. In the latter case, an isolated colony of the indicator strain was inoculated in 5 ml of LB and incubated at 37°C under shaking to an OD_600_ of 0.4 (≈4x10^8^ CFU/ml). Subsequently, an aliquot of phage suspension was added to obtain a multiplicity of infection (MOI), i.e., a phage/host ratio, of 0.01. This suspension was incubated at 37°C in aerobic conditions for 4 hours and then centrifuged at 1,500 x *g* for 15 min. The supernatant was filtered at 0.22 µm to remove bacterial debris and titrated by the double-layer agar overlay method as described above. Phage suspensions were subjected to purification by adding 5 ml of polyethylene glycol solution (20% PEG-8000, 3.3 M NaCl) to an iso-volume of phage lysate. The mixture was incubated O/N at 4°C, centrifuged at 12,000 × *g* for 60 min at 4°C and then the pellet was resuspended in 1 ml of SM buffer and stored at 4°C. For electron microscopy analysis, these phage suspensions were further purified from PEG and concentrated (2.5-5 X) by using Amicon Ultra-4 (10 kDa cut off) centrifugal filters (Merck KGaA, Darmstadt, Germany).

### 2.4 Transmission electron microscopy

Purified bacteriophages suspensions were subjected to transmission electron microscopy (TEM) analysis to investigate their morphological features. Samples were diluted 1:10 in distilled water, then processed by negative stain. Briefly, 3.5 μl of each phage suspension (≈10^9^-10^11^ PFU/ml) was laid down on a carbon-coated 300 mesh electron microscopy grid, let to adsorb for 2 minutes and then stained with 1.5% uranyl acetate for 15 seconds. The staining solution was then blotted by filter paper, grid was air-dried, inspected and imaged by TEM. Micrographs were obtained with a FEI Tecnai G2 Spirit TEM microscope (FEI, Eindhoven, The Netherlands) fitted with a TVIPS TemCam F216 CMOS camera (TVIPS, Gilching, German).

### 2.5 Determination of host-range

The host-range of the isolated bacteriophages was evaluated by spot-test using the four indicator strains used for phages isolation plus other 32 clinical isolates of *K. pneumoniae* (ST307, *n* = 12; ST147, *n* = 10; ST258/512, *n* = 4; ST101, *n* = 1; ST395, *n* = 5). In these experiments, a well-isolated colony of each bacterial strain was inoculated into 500 μl of LB and incubated under shaking at 37°C for 30 min. Aliquots of 10 μl of each inoculum were spotted onto an LBA plate, allowed to absorb and then challenged with 5 μl of phage suspensions (10^8^ PFU/ml) by deposition with partial overlap to the bacterial spot. Results were recorded after 5-6 hours of incubation at 37°C by observation or not of a lysis halo in the overlapped area. Each strain was finally classified as “sensitive (++)”, if the lysis zone was clear, “partially sensitive (+/-)”, if the lysis zone was present but not well defined, and “resistant (-)”, in absence of lysis.

### 2.6 Efficiency of plating

The ability of the isolated phages to produce productive infections on isolates displaying a “sensitive” or “partially sensitive” phenotype by spot-test, was assessed through the determination of the efficiency of plating (EOP) using the double-layer agar overlay method, as previously described (Khan Mirzaei and Nilsson, 2015). In brief, 5 μl of tenfold serial dilution of phage suspensions (range 10^8^–10^3^ PFU/ml) were spotted on a bacterial lawn of each tested strain. Plates were incubated O/N at 37°C and the number of plaque-forming units (PFU) was finally enumerated. Assays were performed in triplicate for each phage/strain combination and results were computed as the mean of three observations. The EOP was calculated as the ratio between PFU/ml on the tested strains and the PFU/ml on the corresponding indicator strain and used to rank lytic efficiency as “high productive” (EOP ≥ 0.5), “medium productive” (0.1 ≤ EOP < 0.5), “low productive” (0.001 ≤ EOP < 0.1), “inefficient” (EOP < 0.001), and “lysis from without” (in case of observation of lysis but lacking of distinct lysis plaques in any tested concentrations).

### 2.7 One-step growth curve

The latency period and the burst size of the isolated phages were investigated by using the one-step growth curve technique, as previously described (Kropinski, 2018). Briefly, a single well-isolated colony of each indicator strain was inoculated into 5 ml of LB and incubated under aerobic conditions at 37°C to OD_600_ = 0.3-0.4. One ml of each culture was centrifuged at 16,873 x *g* for 5 min and the pellet resuspended in 1 ml of SM buffer. Bacterial suspensions were mixed with phage lysates to achieve a MOI of 0.01, let stand for 5 or 10 min (depending on the phage and according to preliminary tests) at 37°C statically in a water-bath to allow phage absorption, and then centrifuged at 16,873 x *g* for 2 min at RT to remove the non-absorbed phages. The pellet was then suspended in 1 ml of SM buffer, diluted 1:1000 in 10 ml of LB medium and incubated at 37°C under shaking. Aliquots of 100 µl were sampled every 5 or 10 min up to 50 or 120 minutes. Each aliquot was mixed with 5 ml of soft agar, poured onto an LBA plate and incubated O/N at 37°C. The latency period was defined as the interval between the onset of infection (excluding the pretreatment period) and the beginning of the release of phage progeny. The burst size of each phage was calculated as the ratio between the average number of released phage particles at plateau and the average number of infected bacterial cells during the latency period. Each experiment was performed three times, and results are reported as mean values ± standard deviation.

### 2.8 Effects of pH and temperature on phage stability

To evaluate the stability to variations of environmental conditions, phages were incubated at different temperatures or pH. Thermal stability was assessed by incubating 1 ml of phage suspensions (range 10^8^-10^9^ PFU/ml) at 37°C, 50°C, 60°C, and 70°C for 10, 20, 40 and 60 minutes. Stability to pH was evaluated by diluting 10 μl of each phage suspension (range 10^7^-10^10^ PFU/ml) 1:100 in 1 ml of SM buffer previously adjusted to pH from 2.0 to 12.0 with intervals of 1 unit. Mixtures were then incubated statically at 37°C for 60 min. In both tests, at each time point serial dilutions of phages were titrated using the corresponding indicator strain by double-layer agar assay. Both experiments were performed in triplicate and results are reported as mean values ± standard deviation.

### 2.9 Phage DNA extraction and genome sequencing

The Wizard^®^ DNA Clean-Up System (Promega, Madison, WI, USA) was used for extraction of bacteriophage genomic DNA, following the protocol provided by the manufacturer. Before DNA extraction phage lysates were treated by adding 0.8 μl of DNase I (2 U/μl) and 1 μl of RNase A (100 mg/ml) and incubated at RT for 15 min to remove host nucleic acids. The obtained DNA preparations were quantified by using a NanoDrop Spectrophotometer (Nanodrop Technologies Inc., Wilmington, USA) and visualized by 0.8% agarose gel electrophoresis to check the integrity of the genome. Whole genome sequencing was performed by Illumina NovaSeq 6000 platform with a paired end (2 x 150 bp) approach by using the Illumina DNA Prep kit (Illumina Inc., San Diego, CA, USA).

### 2.10 Bioinformatics analysis

Raw sequencing reads were quality checked with the software FastQC (version 0.11.9) and assembled *de novo* by using the Shovill pipeline (version 1.1.0) and SPAdes (version 3.15.5) as assembler. The obtained contigs were analyzed with PhageTerm (version 1.0.11) to derive information on each genome topology, tentatively define each phage DNA termini and DNA packaging models (Garneau et al., 2017). Search for Open Reading Frames (ORFs) and their annotation were performed by using the Rapid Annotation using Subsystem Technologies (RAST) web-service, Prokka (version 1.14.5) and Prodigal (version 2.6.3) (Aziz et al., 2008; Hyatt et al., 2010; Seemann, 2014), followed by manual curation. When different start codons have been proposed, each putative ORFs was manually assessed by searching homologues of the *E. coli* ribosomal binding site (5’- AGGAGG -3’) upstream of the predicted start codons. Putative functions of each ORFs were further verified by using HHpred, HMMER (version 3.3.2), InterPro (version 98.0) and BLASTp (NCBI database) (Finn et al., 2015; Zimmermann et al., 2018; Paysan-Lafosse et al., 2023). Rho-independent transcriptional terminators were identified by using ARNold, TransTermHP and PePPER web-software (Kingsford et al., 2007; Naville et al., 2011; de Jong et al., 2012), while putative promoters were searched by using PhagePromoter (Sampaio et al., 2019). Only transcriptional terminators detected by at least two tools and promoters displaying a score >0.8 and not fully located on ORFs were considered. Search of polysaccharide depolymerase encoding genes was performed by using DepoScope (Concha-Eloko et al., 2024). The BLASTn tool (https://blast.ncbi.nlm.nih.gov/Blast.cgi) was used to search for the closest homologs of each isolated phage among those deposited in databases of the International Nucleotide Standard Database Collaboration (INSDC). Genomes displaying >70% nucleotide identity (computed as % identity multiplied by % coverage) over their full genome length were retrieved and used for phylogenetic analysis and taxonomic classification (Turner et al., 2021). The VICTOR webserver and VIRIDIC were employed to create phylogenetic trees and classify the viruses, respectively (Meier-Kolthoff and Göker, 2017; Moraru et al., 2020).

## 3 Results

### 3.1 Isolation of *K. pneumoniae* phages

**Figure 1:**
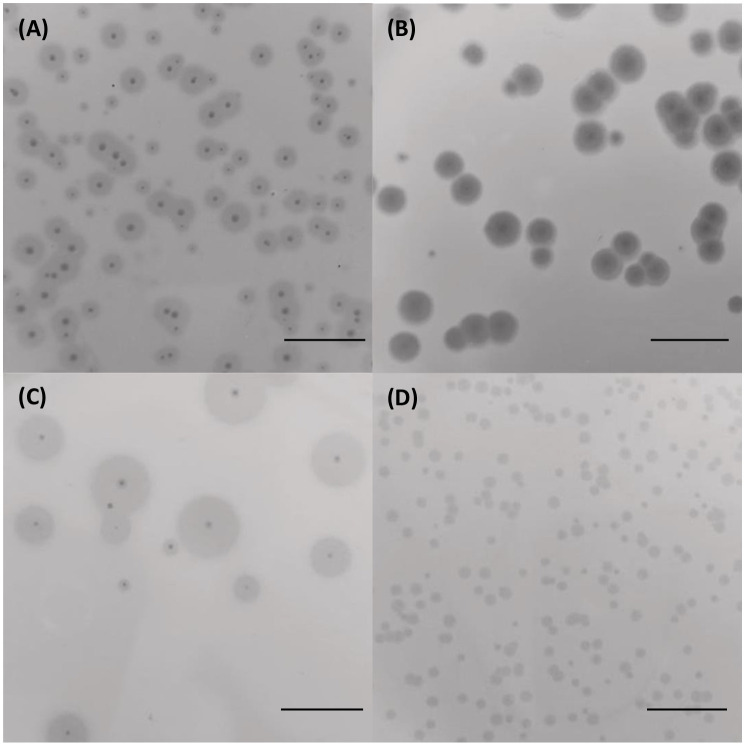
Morphology of lysis plaques formed by the four bacteriophages on their indicator strains. Plaques of GP-1 on *K*. *pneumoniae* EuSCAPE_IT395 **(A)**, GP-5 on *K*. *pneumoniae* KP411 **(B)**, GP-2 on *K*. *pneumoniae* KP263 **(C)** and GP-4 on *K*. *pneumoniae* KP20-LU **(D)**. The bar indicates 1 cm.

Four distinct bacteriophages, designated as vB_KpS_GP-1, vB_KpP_GP-5, vB_KpP_GP-2, and vB_KpP_GP-4, were isolated from wastewater samples using *K. pneumoniae* strains EuSCAPE_IT395 (ST307), KP411 (ST307), KP263 (ST147), and KP20LU (ST147), respectively. The four phages, named in accordance with directions proposed by Turner et al., are hereafter referred to as GP-1, GP-5, GP-2, and GP-4 for short (Turner et al., 2021). All the bacteriophages formed small, transparent, and well-evident plaques with varying diameters on their respective host strains. Plaques of all phages exhibited a lysis zone with a diameter of ≈ 1 mm. In addition, plaques formed by GP-1, GP-2, and GP-5 were surrounded by an evident fuzzy halo of varying opacity and diameters (**Figure 1**).

### 3.2 Phage host range and EOP

The host range of the isolated phages was assessed by testing a collection of 32 MDR *K. pneumoniae* strains plus the four indicator strains used for phage isolation (**Table 2**). Results showed that GP-1 was able to selectively lyse, although with different efficiency, 10/13 (77%) strains of *K. pneumoniae* ST 307 (excluding its indicator strain) and that GP-5 was able to perform a productive infection on two *K. pneumoniae* ST307 strains both resistant to the infection by GP-1. Interestingly, GP-5 was also able to infect, even if at a low productivity, a capsule-deficient strain of *K. pneumoniae* ST258 clade II resistant to the φBO1E phage (D’Andrea et al., 2017; Henrici De Angelis et al., 2021). A similar association between ST and phage host-range was observed with GP-4, which efficiently lysed 10/11 (91%) strains of *K. pneumoniae* ST147 (excluding its indicator strain), while GP-2 was able to productively infect 3 ST147 strains, including the strain resistant to GP-4. In addition, the GP-2 phage was also able to infect with low or inefficient productivity 9/14 (64%) strains of ST307 (**Table 2**). Notably, except for GP-5, the four phages were not able to infect strains of ST different from ST147 or ST307.

**Table 2:** Host range of the four phages on 36 MDR *K. pneumoniae* strains as determined by spot-test and EOP analysis.

Strain	ST	Carbapenemase	*wzi*, K locus	GP-1	GP-5	GP-2	GP-4	Reference
EuSCAPE_IT076	307	ND	*wzi173*, KL102	0.0003 (INEF)	Lfw	R	R	David et al., 2019
KP320	307	KPC-3	*wzi173*, KL102	1.09 (HP)	Lfw	0.002 (LP)	R	Di Pilato et al., 2021
EuSCAPE_IT395	307	ND	*wzi173*, KL102	1.00^I^ (HP)	R	0.001 (LP)	R	David et al., 2019
EuSCAPE_IT377	307	ND	*wzi173*, KL102	0.56 (HP)	R	0.001 (LP)	R	David et al., 2019
EuSCAPE_IT396	307	KPC-2	*wzi173*, KL102	0.71 (HP)	R	0.00008 (INEF)	R	David et al., 2019
KP388	307	KPC-2	*wzi173*, KL102	0.59 (HP)	R	0.00009 (INEF)	R	Di Pilato et al., 2021
EuSCAPE_IT068	307	KPC-3	*wzi173*, KL102	0.88 (HP)	R	0.00013 (INEF)	R	David et al., 2019
KP305	307	KPC-2	*wzi173*, KL102	0.49 (MP)	Lfw	0.00008 (INEF)	R	Di Pilato et al., 2021
EuSCAPE_IT400	307	ND	*wzi173*, KL102	R	1.27 (HP)	R	R	David et al., 2019
EuSCAPE_IT113	307	ND	*wzi173*, KL102	0.66 (HP)	R	0.00013 (INEF)	R	David et al., 2019
KP357	307	KPC-2,	*wzi173*, KL102	R	R	R	R	Di Pilato et al., 2021
KP259	307	KPC-3 and OXA-48	*wzi173*, KL102	0.04 (LP)	R	R	R	Di Pilato et al., 2021
KP416	307	KPC-2	*wzi173*, KL102	0.73 (HP)	R	0.00012 (INEF)	R	Di Pilato et al., 2021
KP411	307	KPC-2	*wzi173*, KL102	R	1.00 ^I^ (HP)	R	R	Di Pilato et al., 2021
KP263	147	KPC-3	*wzi64*, KL64	R	R	1.00 ^I^ (HP)	0.58 (HP)	Di Pilato et al., 2021
KP267	147	KPC-3	*wzi64*, KL64	R	R	1.47 (HP)	1.50 (HP)	Di Pilato et al., 2021
Kpn 293359	147	KPC-3	*wzi64*, KL64	R	R	0.10 (MP)	R	NA
KP-14442FI	147	NDM-1	*wzi64*, KL64	R	R	Lfw	1.71 (HP)	Di Pilato et al., 2022
KP-20LU	147	NDM-1	*wzi64*, KL64	R	R	Lfw	1.00 ^I^ (HP)	Di Pilato et al., 2022
KP-2LU	147	NDM-1	*wzi64*, KL64	R	R	Lfw	0.89 (HP)	Di Pilato et al., 2022
KP-25PA	147	NDM-1	*wzi64*, KL64	R	R	Lfw	0.93 (HP)	Di Pilato et al., 2022
KP-21POB	147	NDM-1	*wzi64*, KL64	R	R	Lfw	1.03 (HP)	Di Pilato et al., 2022
KP-37LI	147	NDM-1	*wzi64*, KL64	R	R	Lfw	0.90 (HP)	Di Pilato et al., 2022
22385	147	NDM-1	*wzi64*, KL64	R	R	Lfw	1.12 (HP)	Coppi et al., 2022
21491	147	NDM-1	*wzi64*, KL64	R	R	Lfw	0.91 (HP)	Coppi et al., 2022
21376	147	NDM-1	*wzi64*, KL64	R	R	Lfw	1.39 (HP)	Coppi et al., 2022
KKBO-1	258	KPC-3	*wzi154*, KL107	R	R	R	R	Cannatelli et al., 2013
BO-FR-1	258	KPC-3	*wzi154*, KL107	R	0.002 (LP)	R	R	Henrici De Angelis et al., 2021
KK207/1	258	KPC-2	*wzi29*, KL106	R	R	R	R	D’Andrea et al., 2014
09C086	512	ND	*wzi154*, KL107	R	R	R	R	Giani et al., 2020
12C47	101	KPC-2	*wzi137*, KL17	R	R	R	R	Giani et al., 2013
KP398	395	OXA-48	*wzi2*, KL2	R	R	R	R	Di Pilato et al., 2021
KP368	395	KPC-3	*wzi2*, KL2	R	R	R	R	Di Pilato et al., 2021
EuSCAPE_IT021	395	ND	*wzi105*, KL108	R	R	R	R	David et al., 2019
KP344	395	KPC-3	*wzi2*, KL2	R	R	R	R	Di Pilato et al., 2021
KP346	395	OXA-48	*wzi2*, KL2	R	R	R	R	Di Pilato et al., 2021

EOP values are reported as the mean of three independent experiments. Indicator strains used for the isolation of a given phage are indicated with I in superscript after the EOP value. Efficiency of lysis is reported and classified as: HP: EOP ≥ 0.5 (high productive); MP: 0.1 ≤ EOP < 0.5 (medium productive); LP: 0.001 ≤ EOP < 0.1 (low productive); INEF: EOP < 0.001 (inefficient); and Lfw: in case of observation of lysis but lacking distinct lysis plaques in any tested concentrations (lysis from without). R, resistant; NA, Not Available; ND, Not Detected.

### 3.3 One-step curve experiments

**Figure 2:**
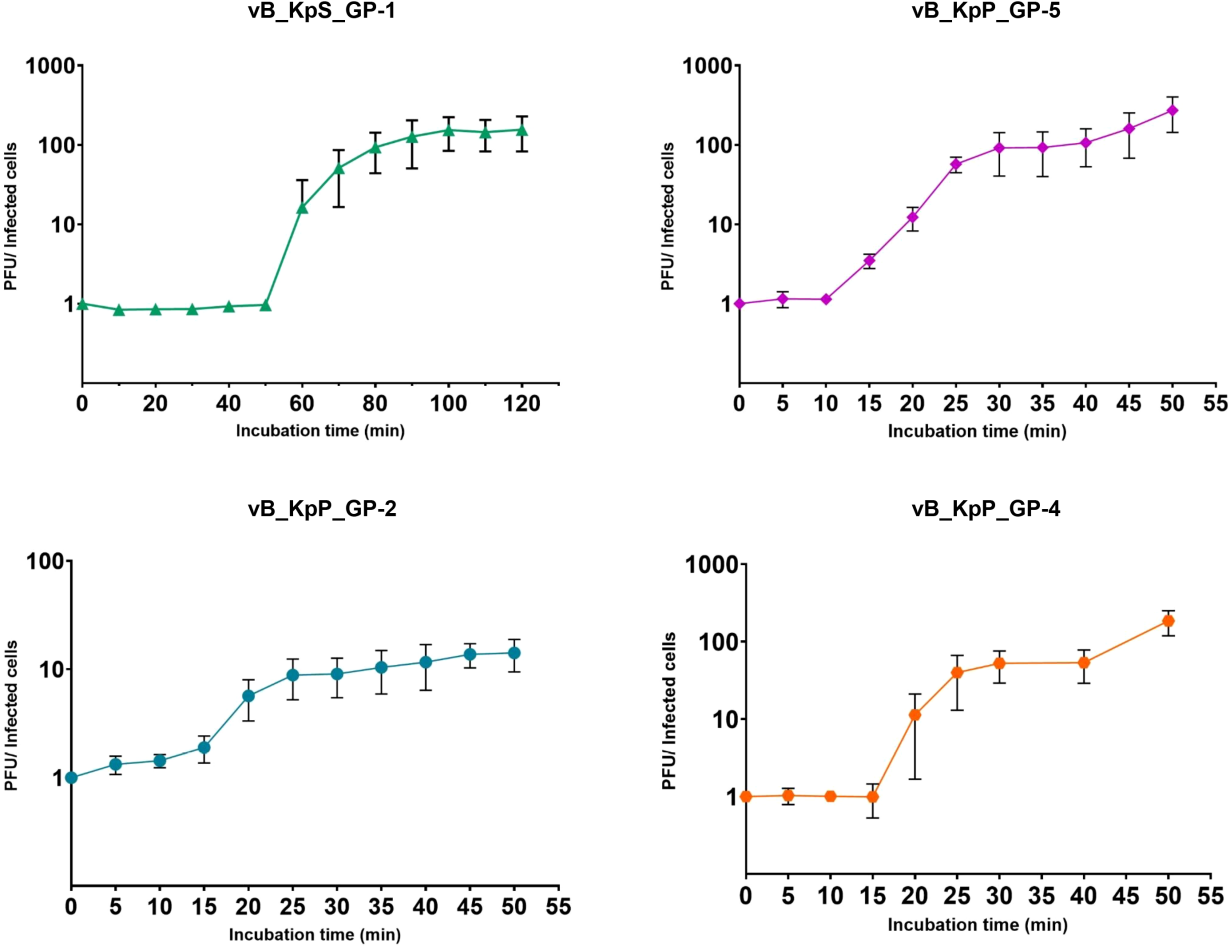
One-step growth curves of the four isolated bacteriophages. The ratios between PFU and the number of infected bacterial cells at different times are shown. Data are the mean from three independent experiments. Vertical black bars indicate one standard deviation.

The assessment of the replicative cycle of the four phages was investigated by one-step growth curve. Results from these experiments showed that phage GP-1 exhibited the longest replicative cycle, approximately 120 minutes, which was characterized by a latent period of ≈50 minutes, a release stage of ≈50 minutes and a burst size of ≈150 PFU/Infected cell. On the contrary, the GP-2, GP-4, and GP-5 phages displayed shorter replicative cycles (from 30 to 40 minutes), were similar to each other in terms of latency periods (from 10 to 15 minutes), and showed burst sizes of 9, 53, and 87, respectively (**Figure 2**).

### 3.4 Sensitivity to temperature and pH variations

**Figure 3:**
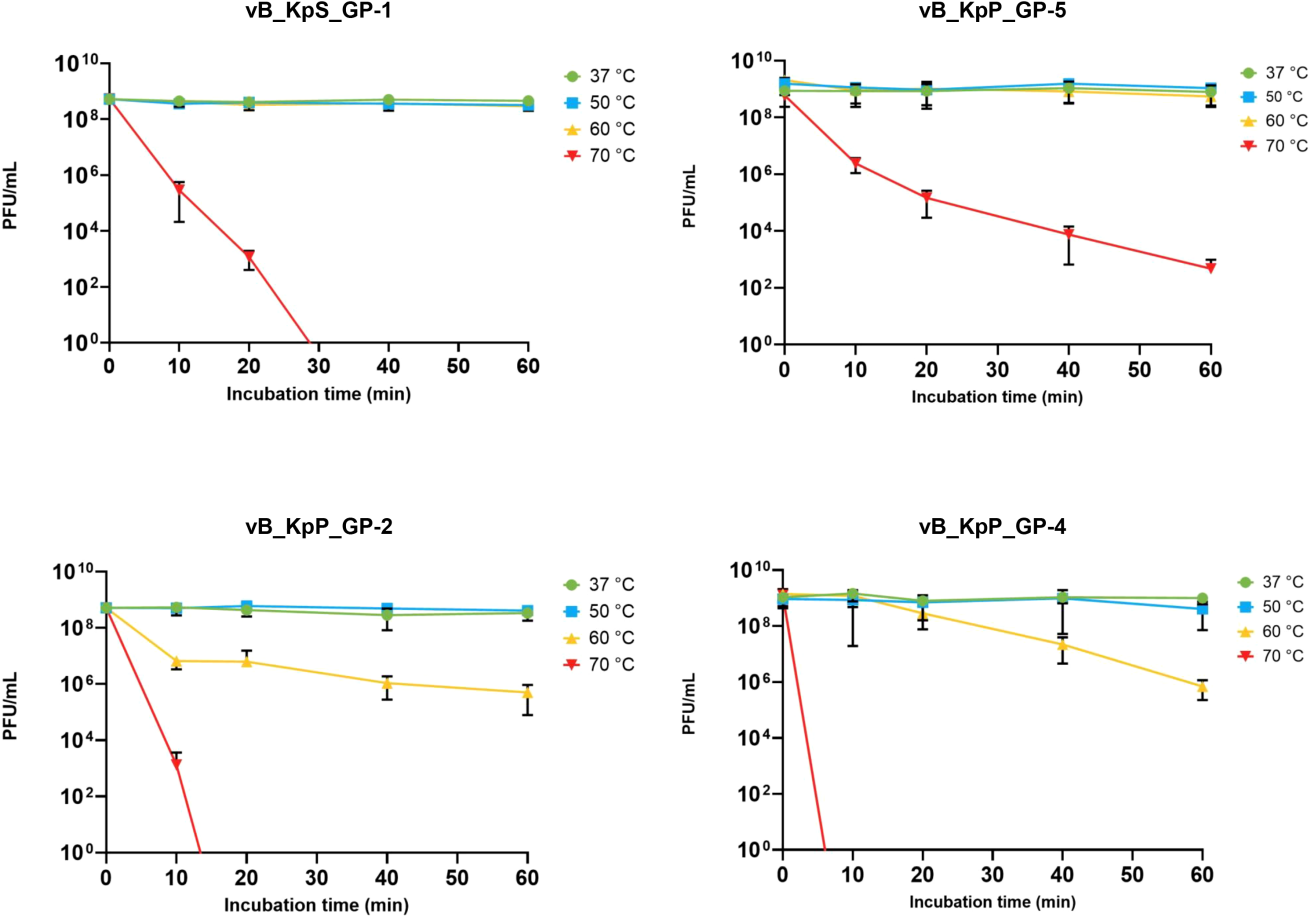
Effect of temperature on infectivity of the four isolated phages. Data represents the mean of three independent experiments. Vertical black bars indicate one standard deviation.

Results from experiments performed to assess the physical and chemical stability of the four phages, showed that GP-1 and GP-5 were stable after 1-hour incubation at temperatures ≤60°C, with a notable decrease of phage titers only after incubation at 70°C. In detail, 30 minutes at 70°C were able to reduce the GP-1 infectivity to zero, while GP-5 demonstrated a higher stability, showing a ≈6-log loss of infectivity after 60 minutes of incubation at the same temperature. On the contrary, a notable decrease of the infective capacity (≥ 3 log) was observed for GP-2 and GP-4 after incubation for 1 hour at 60°C, with a total loss of infectivity following 20- or 10-minutes incubation at 70°C, respectively (**Figure 3**).

**Figure 4:**
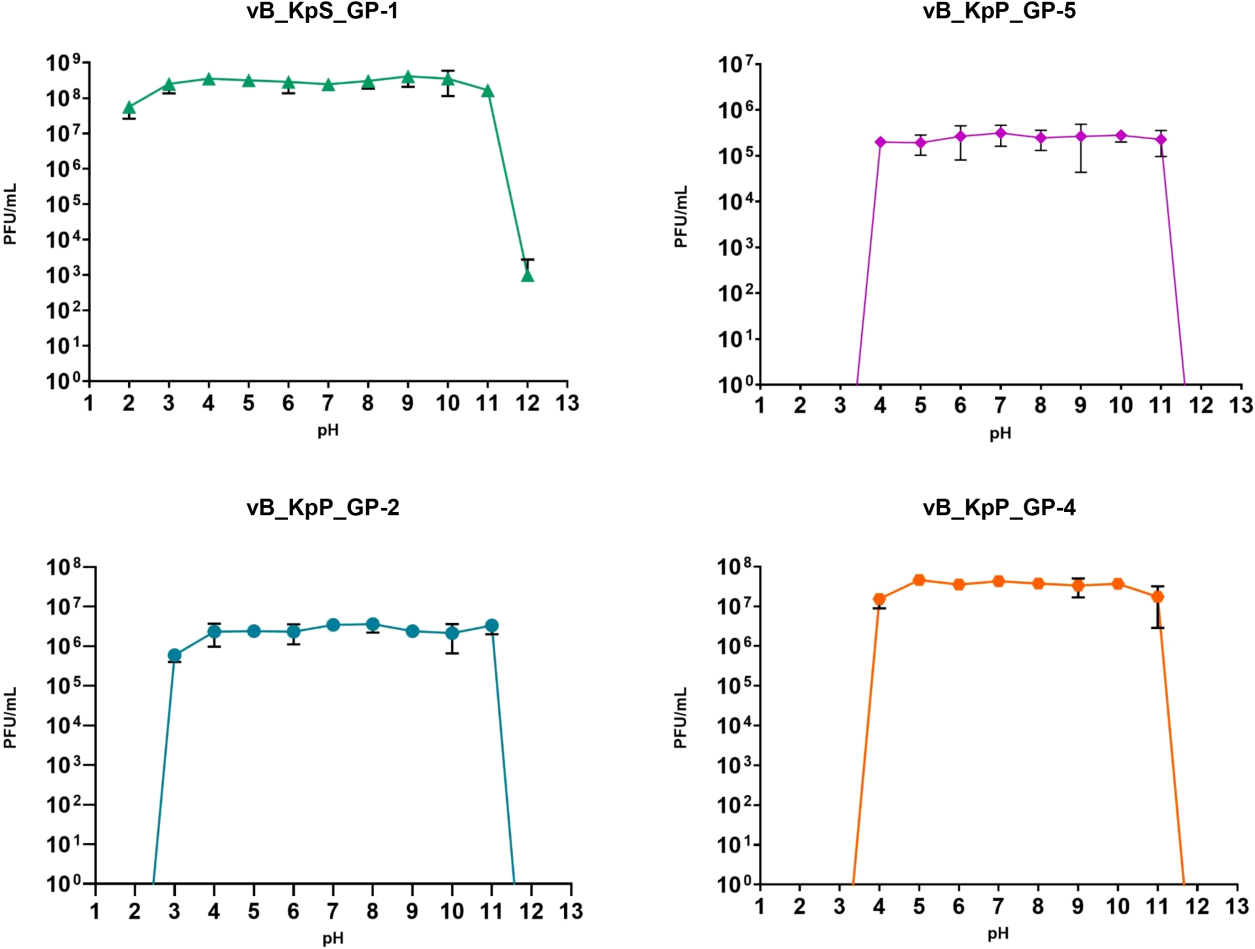
Influence of pH towards phages infectivity. Data are the mean of three independent experiments. Vertical black bars represent one standard deviation.

Experiments to assess the effects of pH on phage infectivity demonstrated that all four bacteriophages exhibited comparable stability with limited loss of infective ability within the pH range of 4 to 11. In addition, GP-2 was overall stable also after 1-hour incubation at pH 3 and GP-1 displayed the highest pH stability range, revealing a notable loss of phage infectivity only after 60 minutes of incubation at pH 12 (**Figure 4**).

### 3.5 Transmission electron microscopy

**Figure 5:**
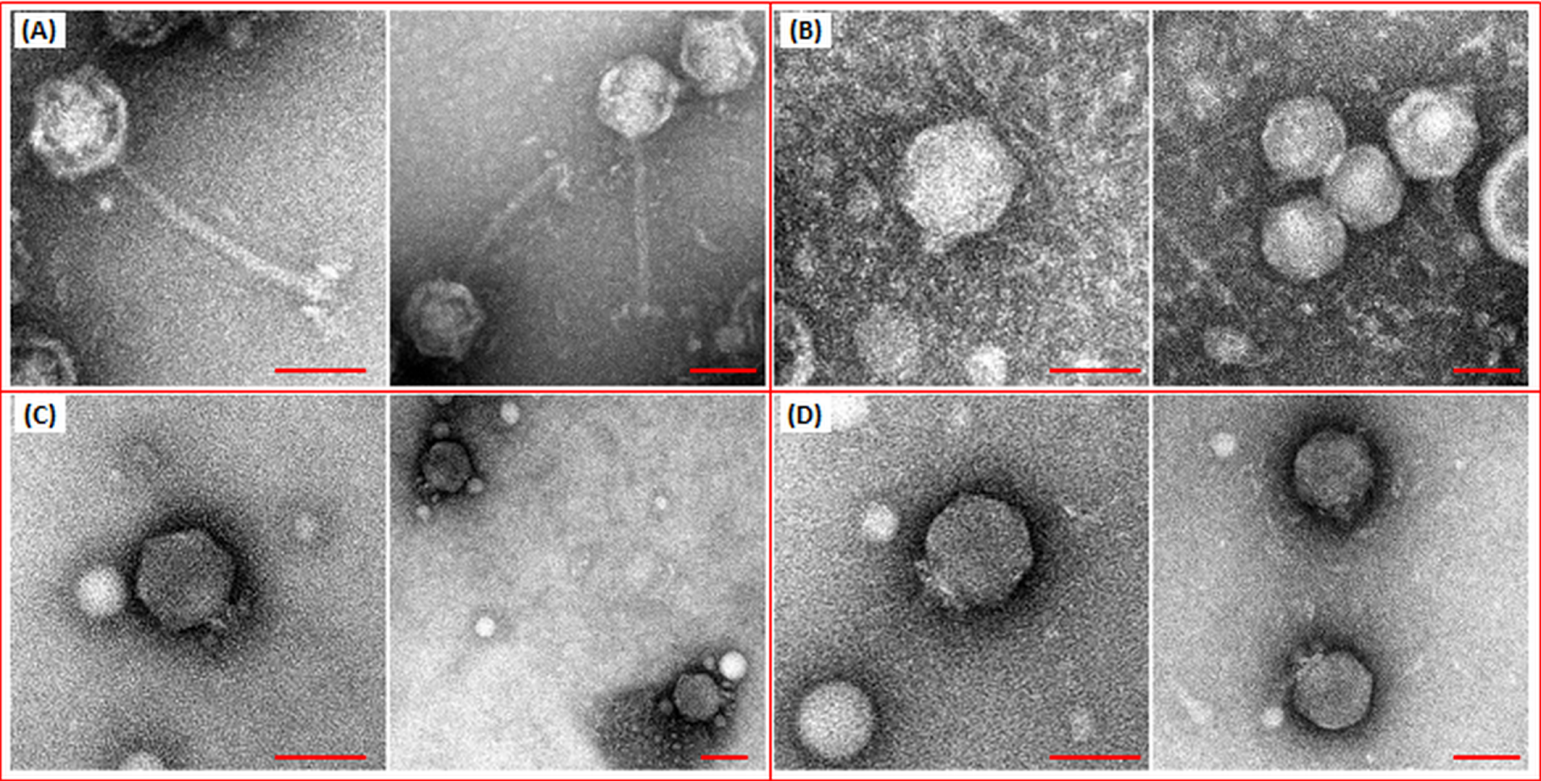
Transmission electron microscopy micrograph of phages vB_KpS_GP-1 **(A)**, vB_KpP_GP-5 **(B)**, vB_KpP_GP-2 **(C)**, and vB_KpP_GP-4 **(D)**. The bar indicates 50 nm.

TEM analysis showed that all phages belonged to the *Caudoviricetes* class. In detail, GP-1 exhibited typical features of siphovirus, composed of an icosahedral capsid with a diameter of ≈54 nm and a long, non-contractile and flexible tail of ≈130 nm. On the contrary, GP-2, GP-4, and GP-5 were characterized by a short, non-contractile tail connected to an icosahedral capsid with a diameter of ≈54 nm, ≈59 nm, and ≈60 nm, respectively. Overall, the observed morphological features allowed the classification of the latter three phages as podoviruses (**Figure 5**).

### 3.6 Bioinformatics analysis

The whole genomes of the isolated phages were obtained by using a Next Generation Sequencing approach. Results from *de novo* assembly, followed by PhageTerm analysis, revealed that all the four genomes were composed by double-stranded DNA and showed similar sizes and GC content (**Table 3**). A total of 48-50 ORFs, all encoded on the same strand, were identified in the three podoviruses (GP-2, GP-4, and GP-5), while 81 ORFs, encoded on either strand, were present in the siphovirus GP-1. Results from the pipeline employed for genome annotation allowed to infer a putative function for 39.1% (GP-1), 84.0% (GP-2), 58.3% (GP-4), and 84.0% (GP-5) ORFs. The genomes of the three podoviruses were characterized by direct terminal repeats located at the 5′- and 3′-ends, with sizes of 179 bp (GP-2), 239 bp (GP-5), and 584 bp (GP-4), while the genome of GP-1 was circularly permuted, suggesting a headful-packaging mechanism in which cutting at different positions of linear DNA concatemers occurs during phage assembly until the capsid is entirely filled with genetic material.

**Table 3:** Morphology and genomic features of the isolated phages.

Phage	Morphology	Genome size (bp)	GC %	N° of ORF	Hypothetical proteins	DNA metabolism-related proteins	Structural proteins	Host lysis	Proteins with other functions
GP-1	siphovirus	47,648	48.3	81	49	8	12	3	9
GP-2	podovirus	41,109	52.7	50	8	17	13	3	9
GP-4	podovirus	43,687	55.8	48	20	14	11	3	0
GP-5	podovirus	39,069	50.6	50	8	14	13	3	12

Search for regulatory elements revealed the presence of two putative rho-independent terminators in the genomes of GP-2, GP-4, and GP-5, while ten were identified in GP-1. In addition, one (GP-4) to five (GP-5) host promoters have been detected and 14 and 15 phage promoters, showing high similarity to the *E. coli* phage T7 and T3 RNA polymerases binding sites, were annotated in GP-2 and GP-5, respectively (**Supplementary Table 1**; **Supplementary Figures 1**, **2**).

No genes associated with virulence factors, antibiotic resistance, or lysogeny were found. The lack of ability to lysogenize its indicator strain was also experimentally confirmed by a PCR approach for the siphovirus GP-1 by using ten different phage-resistant derivatives obtained after infections at high MOI (data not shown). Search for polysaccharide depolymerase-encoding genes suggested the presence of one hit in the GP-1, GP-4, and GP-5 phages (CDS 58, CDS 35, and CDS 39, respectively) and of two hits in the GP-2 genome (CDS 40 and CDS 45).

### 3.7 Phylogenetic analysis

Results from phylogenetic analysis suggested that GP-1 was a member of a novel genus of the *Caudoviricetes* class. Indeed, BLAST analysis showed that the GP-1 genome displayed values >70% only with the VLCpiS13a (NC_071152.1) phage (Beamud et al., 2023) and VIRIDIC confirmed these findings. Similar results were obtained for GP-4, for which both BLAST and VIRIDIC analysis suggested that it was part of a new genus together with the vB_Kpl_K59PH2 (OY757063.1), 6937 (OL362270.1), and KYP (ON755176.1) phages. Conversely, results of taxonomic analysis of GP-2 and GP-5 obtained from the two approaches suggested that these phages were members of the *Przondovirus* and *Teetrevirus* genus, respectively. Phylogenetic trees obtained by VICTOR analysis are reported in **Supplementary Figures 3**-**6**.

## 4 Discussion

The increasing prevalence of antibiotic-resistant bacterial strains in clinical environments (including both acute care and long-term care rehabilitation facilities) poses significant and worrying public health issues on a global scale. In particular, the rapid dissemination of high-risk CR-Kp clones in these settings strongly limits the available therapeutic options. Therefore, the development of alternative therapies that can replace or complement the use of conventional antibiotics is a major and unmet need. In this context, lytic bacteriophages, or their components, may represent effective tools to address this problem.

Here we report on the isolation and characterization of four newly discovered lytic bacteriophages targeting two major clones of CR-Kp (ST147 and ST307) circulating at a worldwide scale. In detail, to develop a phage cocktail able to lyse the majority of clinical isolates of *K. pneumoniae* ST307 and ST147, the strains KP411 and KP20LU, resistant to the lytic activity of GP-1 and GP-2, were used as hosts for the isolation of GP-5 and GP-4, respectively. Our results demonstrated that, in combination of two, the described bacteriophages displayed selective lytic capacity against almost all tested clinical isolates belonging to the two clones of interest, with remarkable efficiency. Resistance to phage lysis by some tested strains could be due to the extensive intraclone diversification of the capsular polysaccharide genetic loci commonly observed in *K. pneumoniae* clinical isolates, as previously reported for members of the CG258 (Wyres et al., 2015).

All the characterized phages exhibit a strictly lytic nature and feature a very narrow host range, aspects that can be particularly useful to select candidates for targeted phage-cocktails. Indeed, with the exception of GP-2 and to a lesser extent GP-5, each phage was able to give productive infections only on strains characterized by a given capsular polysaccharide genetic locus, suggesting that the bacterial target exploited at the initial stages of phage infection is a component of the capsular polysaccharide. In line with this hypothesis, the ability of GP-2 to lyse with low productivity strains of ST307 could be related to the presence in its genome of two capsular depolymerase encoding genes. Moreover, three phages produce plaques surrounded by prominent and fuzzy halos, thus underscoring the likely production of polysaccharide depolymerases. The selectivity to lyse only pathogens responsible of an infection, while leaving the common microbial flora unaffected, is one of the main advantages of using phage therapy or phage-based drugs. Capsular depolymerases could be components of novel biotechnological drugs able to degrade biofilm or enhance the action of host innate immunity against *K. pneumoniae* in a selective manner, as previously demonstrated (Domingo-Calap et al., 2020; Henrici De Angelis et al., 2021). On the other hand, the lytic ability of GP-5 against strains not recognized nor lysed by the other phages (including the previously characterized capsule-deficient derivative of a strain ST258), suggests that the primary receptor for this phage may not be a component of the bacterial capsule. This hypothesis is supported by a bioinformatics analysis, which revealed a high similarity of the entire genomic sequence (95.87%) of GP-5 with the *Klebsiella* phage mtp4 (OX335434.1), which is selective for capsular-deficient strains of *K. pneumoniae* and, similarly to GP-5, exhibits a broader host spectrum (Lourenço et al., 2023). Another point supporting this speculation is given by a genome analysis of the KP411 and EuSCAPE_IT400 strains, in both of which a disruption of the *wbaP* gene, which encodes a glycosyltransferase involved in the first step of the biosynthesis of the capsular polysaccharide, has been detected. Anyway, additional studies on the mechanisms of interaction of the four phages with their hosts through the characterization of phage-resistant derivatives are currently underway.

The phylogenetic and genetic analysis indicated that three of four phages (GP-2, GP-4 and GP-5) are podoviruses from different genera within the *Autographiviridae* family of the class *Caudoviricetes*. Indeed, the genomes of these three phages have some features typical of this family, including: i) the presence of direct terminal repeats at genome ends and genome sizes (39 - 43 Kbp), values similar to those found in the literature for other phages of this family and comparable with the values estimated by the ICTV (Pan et al., 2019; Tan et al., 2019; Li et al., 2021); ii) the presence of genes encoding for RNA polymerase, exploited for an efficient transcription of phage genes; iii) a genetic organization into specific functional modules, with genes encoded on the same DNA strand; and iv) the presence of functional domains typical of polysaccharide depolymerase enzymes in genes of tail fibers. Assignment of GP-2 and GP-5 in the *Przondovirus* and *Teetrevirus* genera, respectively, has been made given their high identity with other phages included in the ICTV. On the other hand, the absence of genomes exhibiting enough similarity to GP-4 or GP-1, together with bioinformatics analysis, suggest that these phages are member of two novel genera. In conclusion, results from this work represent solid basis for further *in vivo* investigations in animal models aimed to elucidate the complex phage/bacterium/host interactions. These preclinical studies will also contribute to evaluate the safety and therapeutic value of the herein reported bacteriophages.

## Data Availability

The nucleotide sequences of vB_KpS_GP-1, vB_KpP_GP-2, vB_KpP_GP-4, and vB_KpP_GP-5 were deposited in the GenBank database under accession numbers PP454752, PP454753, PP454754, and PP454755, respectively.
