# Supplementary material for: Characterization of four novel bacteriophages targeting multi-drug resistant Klebsiella pneumoniae strains of sequence type 147 and 307
Source: Front Cell Infect Microbiol. 2024 Oct 4;14:1473668. doi: 10.3389/fcimb.2024.1473668 (PMC11486747; doi:10.3389/fcimb.2024.1473668)
Supplement: Supplementary file 1 [file DataSheet1.docx]

Supplementary Material

# Supplementary Figures and Tables

## Supplementary Tables

Supplementary Table 1. Transcription regulatory genetic elements present in the genome of the four phage isolates.

| Phage | Element  type | Start position | End Position | Strand | Sequence (5’-3’) |
| --- | --- | --- | --- | --- | --- |
| GP-1 | Host promoter | 1820 | 1846 | + | AAAGATTAACCATGACCATGCTATAAT |
| GP-1 | Rho independent terminator | 1835 | 1876 | + | CCATGCTATAATCCCATCCATGCGATGGGATTTTTTATGGTG |
| GP-1 | Rho independent terminator | 4242 | 4280 | + | CGCACAAGAAAGCCGCCTGATGGCGGCTTAATTTTATCT |
| GP-1 | Rho independent terminator | 8090 | 8127 | + | GCAATAAAAAAGCCCCGTAAAGGGGCTTTGTTTTACAG |
| GP-1 | Rho independent terminator | 14461 | 14497 | + | GTAAAAATAAAGGGGCGAAAGCCCCTTTTAACTATCA |
| GP-1 | Rho independent terminator | 15070 | 15108 | + | AGTAAGATAAACCCGCAAACAAGCGGGTTTTTTATTGGG |
| GP-1 | Rho independent terminator | 15938 | 15961 | - | AAAGCCACTTCCGGGGCTTTTCTT |
| GP-1 | Rho independent terminator | 21710 | 21754 | + | CGGCATAAAGAAAACCCCCGAAAGGGGGTTTATTTTTTCAGCAAT |
| GP-1 | Host promoter | 23063 | 23092 | - | TTGACAGGAGACACAAGACTGACGTAGATT |
| GP-1 | Rho independent terminator | 34315 | 34356 | + | CCCAGTTGAAAAAGCCCCCGATGGGGCTTTTTCACTTATCAA |
| GP-1 | Rho independent terminator | 40247 | 40285 | + | TAAAAAACAAACCCTCCTGATGGAGGGTTTTTGTTATCG |
| GP-1 | Rho independent terminator | 45549 | 45585 | + | CCACCCTTTAACCCGCTTCGGCGGGTTCTTTTTTTGC |
| GP-2 | Phage promoter | 329 | 351 | + | AATTAGGTATCACTATAAGGAGA |
| GP-2 | Host promoter | 430 | 458 | + | TTGACATTAGGTAACAGCTATGGTCTAAT |
| GP-2 | Phage promoter | 5894 | 5916 | + | TAATTAGGTAGCACTATAGGGAA |
| GP-2 | Phage promoter | 6542 | 6564 | + | AATAAGGTAGCACTATAGGAACA |
| GP-2 | Phage promoter | 7068 | 7090 | + | GATTAGGTAGCACTATAGGCAGA |
| GP-2 | Rho independent terminator | 7081 | 7131 | + | TATAGGCAGACTCAAGGTCATCGGATTCCGGTGGCCTTTATGATTGCTTAT |
| GP-2 | Phage promoter | 8583 | 8605 | + | AATTAGGTAGCACTATAGGAGAC |
| GP-2 | Phage promoter | 9759 | 9781 | + | CATTAGGTAGCACTATAGGGACA |
| GP-2 | Phage promoter | 11429 | 11451 | + | ACATTAGGTAGCACTACAGGGAG |
| GP-2 | Phage promoter | 18222 | 18244 | + | AATTAGGTAGCACTATAGGGAAG |
| GP-2 | Host promoter | 18534 | 18559 | + | TTGATTATGTGTTTCTCACCTAAGAT |
| GP-2 | Phage promoter | 20653 | 20675 | + | AATTAGGTAGCACTATAGGGAGA |
| GP-2 | Phage promoter | 21730 | 21752 | + | AATTAGGTAGCACTATAGGGAGA |
| GP-2 | Rho independent terminator | 23320 | 23377 | + | AAACTAGCCAAACCCCTTGGGGACCACTCACGGTCTCTGAGGGGTTTTTCGTTAGGAG |
| GP-2 | Phage promoter | 26829 | 26851 | + | AATTAGGTAGCACTATAGGGAGA |
| GP-2 | Phage promoter | 34115 | 34137 | + | AATTAGGTAGCACTATAGGGAGA |
| GP-2 | Host promoter | 37417 | 37448 | + | TTGACTGGAAGAAAGCTAATAAGGAGTAACAT |
| GP-2 | Phage promoter | 40433 | 40455 | + | AATTAGGTAGCACTATAGGGAGA |
| GP-2 | Phage promoter | 40570 | 40592 | + | CACTAAGAGCCAACATAAGGAGG |
| GP-4 | Host promoter | 1548 | 1576 | + | TTGACAAGGATAACTCTTAGCGCTAGATT |
| GP-4 | Rho independent terminator | 4494 | 4534 | + | GTTTGCCGAAAGGCCTGCACTCGCGGGCCTTTTCTCAATAT |
| GP-4 | Rho independent terminator | 25128 | 25172 | + | TAAGACCAACTGGCCTGCCCTTATGGGTGGGCCTTTTTTCGTTGA |
| GP-5 | Phage promoter | 393 | 415 | + | CATTAACCCTCACTAAAGGGAAT |
| GP-5 | Host promoter | 469 | 498 | + | TTGACTTTAAGTAACCCTTAAGGCTATTAT |
| GP-5 | Host promoter | 597 | 626 | + | TTGACAACATGAAGTAACATGCAGTAAGAT |
| GP-5 | Host promoter | 703 | 731 | + | TTGACAACATGAAGTAAGCACGGTACGAT |
| GP-5 | Host promoter | 3440 | 3468 | + | TTGACAAGCGTCTACAAGGCTGATAGAGT |
| GP-5 | Phage promoter | 6198 | 6220 | + | GCATTAACCCTCACTAACGGGAG |
| GP-5 | Phage promoter | 6542 | 6564 | + | AGTTAACCCTAACTAACGGGAGA |
| GP-5 | Phage promoter | 7056 | 7078 | + | TAATAACCCTCACTAACAGGAGA |
| GP-5 | Phage promoter | 8587 | 8609 | + | CATTAACCCTCACTAACAGGAGA |
| GP-5 | Phage promoter | 9808 | 9830 | + | TAATTACCCTCACTAAAGGGAAC |
| GP-5 | Phage promoter | 11580 | 11602 | + | AATTAACACTCACTAAAGGGATG |
| GP-5 | Rho independent terminator | 11586 | 11629 | + | CACTCACTAAAGGGATGACATAACGTTGTCCCTTTGTTCTCGCA |
| GP-5 | Phage promoter | 13397 | 13419 | + | AATTAACCCTCACTAACGGGAAC |
| GP-5 | Host promoter | 17348 | 17378 | + | TTGACTTCCTGTGGTGTACCACTGGTAACAT |
| GP-5 | Phage promoter | 17866 | 17888 | + | AATTAACCCTCACTAAAGGGAAG |
| GP-5 | Phage promoter | 20405 | 20427 | + | TAATAACCCTCACTAAAGGGAGA |
| GP-5 | Phage promoter | 21440 | 21462 | + | AATTAACCCTCACTAAAGGGAGA |
| GP-5 | Rho independent terminator | 22943 | 23001 | + | TTACTTTATGAACCCCTTGGGTGCCCTCTGAGGGTATCTGAGGGGTTTTTTGCTTTAAC |
| GP-5 | Phage promoter | 22995 | 23017 | + | CTTTAACCCTCACTAACAGGAGG |
| GP-5 | Phage promoter | 26056 | 26078 | + | GAATTAACCCTCACTAAAGGGAG |
| GP-5 | Phage promoter | 33338 | 33360 | + | AATAAACCCTCACTAAAGGGAGA |
| GP-5 | Phage promoter | 38294 | 38316 | + | GCATTAACCCTCACTAAAGGGAG |

## Supplementary Figures

1 26

T7 --TAATACGACTCACTATAGGGAGA-

T3 --AATTAACCCTCACTAAAGGGAGA-

GP-2 (329-351) --AATTAGGTATCACTATAAGGAGA-

GP-2 (40570-40592) --CACTAAGAGCCAACATAAGGAGG-

GP-2 (7068-7090) --GATTAGGTAGCACTATAGGCAGA-

GP-2 (9759-9781) --CATTAGGTAGCACTATAGGGACA-

GP-2 (11429-11451) -ACATTAGGTAGCACTACAGGGAG--

GP-2 (5894-5916) -TAATTAGGTAGCACTATAGGGAA--

GP-2 (18222-18244) --AATTAGGTAGCACTATAGGGAAG-

GP-2 (6542-6564) --AATAAGGTAGCACTATAGGA-ACA

GP-2 (8583-8605) --AATTAGGTAGCACTATAGGAGAC-

GP-2 (20653-20675) --AATTAGGTAGCACTATAGGGAGA-

GP-2 (21730-21752) --AATTAGGTAGCACTATAGGGAGA-

GP-2 (26829-26851) --AATTAGGTAGCACTATAGGGAGA-

GP-2 (34115-34137) --AATTAGGTAGCACTATAGGGAGA-

GP-2 (40433-40455) --AATTAGGTAGCACTATAGGGAGA-

Consensus AATTAGGTAGCACTATAGGGAGA

**Supplementary Figure 1.** Nucleotide Sequence Alignment of phage promoters detected in the GP-2 genome. Consensus sequences of the T3 and T7 RNA polymerases (Rong *et al.*, 1998) are also shown.

1 24

T7 -TAATACGACTCACTATAGGGAGA

T3 -AATTAACCCTCACTAAAGGGAGA

GP-5 (393-415) -CATTAACCCTCACTAAAGGGAAT

GP-5 (38294-38316) GCATTAACCCTCACTAAAGGGAG-

GP-5 (20405-20427) -TAATAACCCTCACTAAAGGGAGA

GP-5 (9808-9830) -TAATTACCCTCACTAAAGGGAAC

GP-5 (13397-13419) -AATTAACCCTCACTAACGGGAAC

GP-5 (17866-17888) -AATTAACCCTCACTAAAGGGAAG

GP-5 (11580-11602) -AATTAACACTCACTAAAGGGATG

GP-5 (22995-23017) -CTTTAACCCTCACTAACAGGAGG

GP-5 (8587-8609) -CATTAACCCTCACTAACAGGAGA

GP-5 (7056-7078) -TAATAACCCTCACTAACAGGAGA

GP-5 (6198-6220) GCATTAACCCTCACTAACGGGAG-

GP-5 (6542-6564) -AGTTAACCCTAACTAACGGGAGA

GP-5 (26056-26078) GAATTAACCCTCACTAAAGGGAG-

GP-5 (21440-21462) -AATTAACCCTCACTAAAGGGAGA

GP-5 (33338-33360) -AATAAACCCTCACTAAAGGGAGA

Consensus ATTAACCCTCACTAAAGGGAG

**Supplementary Figure 2.** Nucleotide Sequence Alignment of phage promoters detected in the GP-5 genome. Consensus sequences of the T3 and T7 RNA polymerases (Rong *et al.*, 1998) are also shown.

**Supplementary Figure 3.** Phylogenetic tree of the GP-1 phage obtained by VICTOR analysis. Each branch reports the phage name. When name was not available, the phage accession number is reported in parentheses. Phages currently characterized by International Committee on Taxonomy of Viruses are colored according to classification at genus level.

**Supplementary Figure 4.** Phylogenetic tree of the GP-2 phage obtained by VICTOR analysis. Each branch reports the phage name. When name was not available, the phage accession number is reported in parentheses. Phages currently characterized by International Committee on Taxonomy of Viruses are colored according to classification at genus level.

**Supplementary Figure 5.** Phylogenetic tree of the GP-4 phage obtained by VICTOR analysis. Each branch reports the phage name. When name was not available, the phage accession number is reported in parentheses.

**Supplementary Figure 6.** Phylogenetic tree of the GP-5 phage obtained by VICTOR analysis. Each branch reports the phage name. When name was not available, the phage accession number is reported in parentheses. Phages currently characterized by International Committee on Taxonomy of Viruses are colored according to classification at genus level.

Rong, M., He, B., McAllister, W. T., and Durbin, R. K. (1998). Promoter specificity determinants of T7 RNA polymerase. Proc. Natl. Acad. Sci. U.S.A. 95, 515–519.doi: 10.1073/pnas.95.2.515
